# The association between labor epidural analgesia and early-onset postpartum hypertension among parturients with hypertensive disorders of pregnancy: A retrospective cohort study

**DOI:** 10.1371/journal.pone.0325476

**Published:** 2025-08-18

**Authors:** Yongle Li, Jiteng Hu, Jiao Duan, Junjian Wen, Jianxiong Huang, Jie Shen, Zurong Hu, Zaisheng Qin

**Affiliations:** 1 Department of Anesthesiology, Nanfang Hospital, Southern Medical University, Guangzhou, Guangdong, China; 2 Department of Anesthesiology, Guangzhou United Family Hospital, Guangzhou, Guangdong, China; 3 State Key Laboratory of Oncology in South China, Collaborative Innovation Center for Cancer Medicine, Sun Yat-Sen University Cancer Center, Guangzhou, Guangdong, China; 4 Department of Anesthesiology, Guangdong Women and Children Hospital, Guangzhou, Guangdong, China; Universidad de Murcia, SPAIN

## Abstract

**Background:**

This study aimed to investigate the association of different durations of labor epidural analgesia (LEA) on early-onset postpartum hypertension (PPHTN) among parturients with hypertensive disorders of pregnancy (HDP).

**Methods:**

We conducted a retrospective cohort study in which patients who were diagnosed with hypertensive disorders of pregnancy between 2018 and 2023. The parturients who received LEA were divided into three groups based on the tertiles of LEA duration: the short-duration group (< 175 minutes), the medium-duration group (175–324 minutes), and the long-duration group (≥ 325 minutes), while parturients who did not receive LEA forming the control group. The primary outcome was early-onset PPTHN, defined as the occurrence of at least one blood pressure measurement meeting hypertensive criteria within 2 days following delivery. Three multivariate logistic regression models was employed to explore the association between the duration of LEA exposure and early-onset PPHTN. Four sets of sensitivity analyses were conducted to assess the robustness of the analysis.

**Results:**

In the study cohort of 1,316 parturients, 36.0% (n = 474) were diagnosed with early-onset PPHTN. Compared with those who did not receive LEA, parturients who received long-exposure LEA had significantly lower incidences of early-onset PPHTN (29.5% vs 41.7%, *P* < 0.05). Univariate survival analysis demonstrated that long-duration LEA was associated with a lower risk of PPHTN during hospitalization (HR = 0.75, 95% CI: 0.59–0.95, *P* < 0.05). All three models showed long-exposure LEA exposure was associated with a reduced incidence of early-onset PPHTN among parturients with HDP. Consistent results were observed in the sensitivity analysis conducted among parturients with documented antepartum hypertension who received multiple antihypertensive medications during pregnancy, as well as among those who did not require antihypertensive therapy during the postpartum period. However, this association did not reach statistical significance when the follow-up period was extended to five days postpartum, or in subgroups of parturients with advanced maternal age, obesity, or PE.

**Conclusion:**

A longer duration of LEA was associated with a reduced risk of early-onset PPHTN among women with HDP. However, this association did not reach statistical significance in subgroups of parturients with advanced maternal age, obesity, or PE.

## Introduction

Although delivery is considered the definitive treatment for hypertensive disorders of pregnancy (HDP), the majority of affected women do not experience immediate resolution of hypertension in the postpartum hospitalization period [1]. Postpartum hypertension (PPHTN) therefore remains a critical yet under-recognized complication. In the United States, 7% of maternal deaths are due to pregnancy- related hypertension, of which 70% occur postpartum [2]. Severe maternal complications, including postpartum eclampsia and stroke may actually common in the early postpartum phase, with most events occurring within 48 hours of delivery [3]. Moreover, women who experience persistent postpartum hypertension during hospitalization face elevated risks of long-term cardiovascular, renal, and cerebrovascular complications [4–6]. These findings highlight the growing recognition of PPHTN as not only an acute postpartum concern but also a marker of future cardiovascular risk, underscoring the need for improved surveillance and management strategies in this population [7].

Epidural analgesia is commonly utilized in women with HDP due to its capacity to provide effective labor analgesia while simultaneously offering hemodynamic stability [8]. Through suppression of the sympathetic nervous system, epidural blockade decreases systemic vascular resistance and attenuates the pressor response to labor, thus reducing the likelihood of acute hypertensive episodes and associated maternal and fetal morbidities [9–11]. Despite these well-characterized intraoperative benefits, the extent to which epidural analgesia influences postpartum blood pressure regulation remains poorly understood. Notably, many local anesthetics used in epidural infusions have extended pharmacokinetic properties, suggesting the possibility of persistent antihypertensive effects beyond delivery. However, current evidence is limited regarding whether these residual effects meaningfully contribute to postpartum blood pressure control or hasten the resolution of hypertension in women with HDP.

Considering that local anesthetics may accumulate in the epidural space during prolonged LEA, potentially leading to delayed recovery from sympathetic blockade, we hypothesized that extended LEA duration may modulate postpartum hemodynamic changes and thereby reduce the risk of early-onset PPHTN. Accordingly, this study aimed to investigate whether different durations of LEA were associated with the risk of early-onset PPHTN, with sensitivity and stratified analyses undertaken to evaluate the consistency and robustness of this relationship across clinically relevant subgroups.

## Materials and methods

### Study design and ethics statement

This study was performed at Guangdong Women and Children Hospital, one of the largest specialized obstetrics and gynecology hospitals in southern China. Ethical approval for this study (No. 202301320) was provided by the Ethical Committee of Guangdong Women and Children’s Hospital. The ethics committee waived the need for informed consent from individual patients in this study because of its retrospective nature. Maternal demographics and clinical data were obtained from medical records via the hospital’s clinical database system. The data were accessed for research purposes on January 6, 2024. All necessary steps were taken to ensure the confidentiality and privacy of the participants, including the anonymization of all personal identifiers prior to analysis.

### Inclusion and exclusion criteria

We conducted a retrospective cohort study involving 2,103 parturients with a live birth via vaginal delivery at ≥28 weeks gestation who were diagnosed with gestational hypertension (GH) or pre-eclampsia (PE) between January 2018 and September 2023. Participants were excluded on the basis of the following criteria: chronic hypertension; severe pregnancy complications such as heart or renal insufficiency; HELLP syndrome; precipitous labor; intrapartum cesarean section; blood loss greater than 500 ml within the first 24 hours postpartum; postpartum severe maternal complications, such as the development of eclampsia, stroke, or other conditions necessitating transfer to the intensive care unit; absence of blood pressure recordings throughout the antenatal period or on the first postpartum day; incomplete medical records, including missing body mass index (BMI) or gestational age; and postpartum hospitalization of less than two days S1 Fig.

### Exposure of LEA

Parturients voluntarily chose to undergo LEA, and for those electing this option, our center implemented a standardized LEA protocol. LEA was routinely initiated at a cervical dilation of approximately 3 cm. LEA was routinely initiated at a cervical dilation of approximately 3 cm. Epidural puncture was performed at L3-4 or L2-3, and an epidural catheter was then inserted 2–3 cm into the epidural space. After confirming the correct placement of the catheter, an initial bolus dose of 8–10 mL of a 1 mg/mL ropivacaine solution combined with 0.5 mcg/mL sufentanil was administered. Following this initial dose, continuous epidural analgesia was provided at a rate of 8–10 mL/hour using the same anaesthetic mixture, delivered via an electronic patient-controlled analgesia (PCA) pump. The PCA pump was set to deliver a patient-controlled dose of 5 mL with a lockout interval of 20 minutes. The analgesic efficacy of LEA was assessed approximately 30 minutes after the initial administration. The goal was to confirm that the visual analogue scale for pain experienced by parturients during contractions remained below 3 and that the highest level of epidural blockade, as determined by thermal sensory testing, did not exceed T8. If the visual analog scale score for pain during labor exceeded 5, an additional 5–10 ml of the same medication was administered. For parturients who did not experience adequate pain relief after this rescue dose, 5 ml of 2% lidocaine was further administered. LEA was routinely discontinued following delivery. Postpartum pain management was standardized, with all patients receiving acetaminophen or diclofenac sodium immediately postpartum, regardless of LEA use. The parturients included in the study were initially divided into two groups on the basis of their receipt of LEA, with parturients who did not receive LEA forming the control group. Due to the lack of definitive literature guidance, we divided participants who received LEA into three groups based on the tertiles of LEA duration: the short-exposure group (< 175 minutes), the medium-exposure group (175–324 minutes), and the long-exposure group (≥ 325 minutes). Consequently, the study included a total of four groups S1 Fig.

### Postpartum hypertension

The primary outcome was early-onset PPTHN, defined as the occurrence of at least one blood pressure measurement meeting hypertensive criteria within 2 days following delivery [12]. Hypertensive values were defined according to standard clinical criteria as systolic blood pressure ≥140 mmHg or diastolic blood pressure ≥90 mmHg. The management of PPHTN in this study was based on the Chinese expert consensus on blood pressure management during pregnancy, which is largely consistent with international guidelines and expert recommendations [13]. Labetalol was the first-line antihypertensive agent, with nifedipine or nicardipine added if blood pressure remained uncontrolled. Women with postpartum blood pressure ≥140/90 mmHg should continue antihypertensive treatment, with the dosage being gradually reduced and eventually discontinued on the basis of the recovery of blood pressure. The frequency of PPHTN, calculated as the ratio of hypertensive episodes to total blood pressure measurements, serves as a supporting metric for evaluating the efficacy of interventions for controlling postpartum hypertension.

### Covariates

The variables collected were selected based on existing literature demonstrating their association with PPHTN [14–16], and included maternal age, body mass index (BMI), parity, gestational age at delivery, presence of diabetes mellitus, type of HDP, oxytocin dose, use of antihypertensive medications, intravenous fluid administration, and estimated blood loss. For analytical purposes, the study period was divided into two distinct clinical phases: the antepartum phase, defined as the interval from hospital admission to the onset of labor, and the postpartum phase, spanning from delivery to hospital discharge. All clinical data were temporally assigned to the corresponding phase based on the documented time of occurrence. Maternal age ≥ 35 years was defined as advanced maternal age. BMI was classificated into normal (< 24.0 kg/m^2^), overweight (24.0–27.9 kg/m^2^), and obesity (≥ 28.0 kg/m^2^). Preterm birth was defined as a gestational age < 37 weeks. Antihypertensive medications included in the analysis were labetalol, nifedipine, and nicardipine. The requirement of these agents was categorized as none, monotherapy, or polytherapy based on documented prescriptions and pharmacy dispensing records.

### Sample size calculation

We hypothesized that longer LEA is associated with a reduced incidence of PPHTN within 48 hours after delivery. Retrospective data indicated that the incidence of PPHTN at within three days was approximately 45% among HDP parturients who did not receive LEA [17]. Considering that about 20% of HDP parturients were anticipated to receive long-duration LEA, we employed PASS 15.0 for calculating the necessary sample size. Our calculations were based on an alpha level (α) of 0.05, a power (1-β) of 0.9, and accounted for a 20% dropout rate, leading to a requirement for a minimum of 908 HDP parturients in our cohort study.

### Statistical analysis

Continuous variables are represented as the mean±SD or median (25th–75th percentiles), depending on their distribution, whereas categorical variables are represented as numbers (percentages). The Shapiro‒Wilk test was used to confirm the normal distribution of continuous variables. Variables were compared within separate subgroups via the Kruskal‒Wallis H test or χ^2^ test, as appropriate. The Bonferroni correction method was used for pairwise comparisons between groups. A univariate Cox proportional hazards model was employed to evaluate the risk of developing PPHTN during hospitalization across varying durations of LEA.

Odds ratios (OR) and 95% confidence intervals (CI) were used to summarize the results of the multivariate logistic regression models. Model 1 was adjusted for basic obstetric and demographic information, including maternal age, BMI, preterm birth, primipara, gestational age. Model 2 was further adjusted for the severity of HDP, by adding the following variables to Model 1: type of HDP, presence of antepartum hypertension, and the antepartum requirement for antihypertensive therapy. Postpartum fluids administration and antihypertensive therapy was added to Model 3 (based on Model 2) to evaluate their potential confounding effect on the association between LEA duration and PPHTN.

Four sets of sensitivity analyses were conducted to assess the robustness of the analysis. First, we aimed to validate the association between longer LEA and early-onset PPHTN among women with antepartum hypertension who required combination antihypertensive therapy. Second, to exclude the potential confounding effect of postpartum antihypertensive treatment, we evaluated this association in a subgroup of participants who did not receive any antihypertensive medication following delivery. Third, we further analyzed whether the association changed when the follow-up period was extended and the primary outcome was redefined as the incidence of PPHTN at five days postpartum. Last, considering that advanced maternal age, parity, obesity, and PE patient are also known risk factors for PPHTN, we conducted stratified analyses according to age, gestational age, parity, obesity status, and type of HDP.

Statistical analyses were conducted via SPSS software version 25. Statistical significance was set at *P* < 0.05.

## Results

### Characteristics of the parturients

Of the 1,316 parturients included in the analysis, 36.0% (n = 474) were diagnosed with PPHTN within two days following delivery, and 74.5% (n = 980) had received LEA during labor. In all three LEA subgroups, none of the parturients reported a Bromage score exceeding 0 during LEA. Additionally, there were no significant differences detected in visual analog scale score at 30 minutes post-LEA and at the time of epidural catheter removal. Similarly, no significant differences were observed in the rates of rescue analgesia required or in the total amount of lidocaine consumed among these groups (Table 1).

**Table 1:** Comparison of demographic characteristics and clinical data among different durations of LEA exposure.

	Duration of LEA				*H*/*χ*^2^	*P*
	None (n = 336)	Short (n = 333)	Medium (n = 332)	Long (n = 315)		
Maternal age, n(%)						
< 35 years	273 (81.3)	267 (80.2)	279 (84.0)	269 (85.4)	3.982	0.263
≥ 35 years	63 (18.7)	66 (19.8)	53 (16.0)	46 (14.6)		
BMI, n(%)						
< 25.0 kg/m^2^	88 (26.2)	60 (18.0)	71 (21.4)	70 (22.2)	9.496	0.148
25.0 ~ 27.9 kg/m^2^	161 (47.9)	171 (51.4)	167 (50.3)	170 (54.0)		
≥ 28 kg/m^2^	87 (25.9)	102 (30.6)	94 (28.3)	75 (23.8)		
Gestational weeks, n(%)						
< 37 weeks	18 (5.36)	26 (7.81)	11 (3.31)	8 (2.54)	11.979	0.008
≥ 37 weeks	318 (94.6)	307 (92.2)	321 (96.7)	307 (97.5)		
Primipara, n(%)	174 (51.8)	174 (52.3)	243 (73.2)^*^	278 (88.3)^*^	135.537	0.000
Diabetes, n(%)	72 (21.4)	82 (24.6)	91 (27.4)	64 (20.3)	5.637	0.131
HDP, n(%)						
Gestational hypertension	197 (58.6)	203 (61.0)	201 (60.5)	189 (60.0)	0.431	0.934
Preeclampsia	139 (41.4)	130 (39.0)	131 (39.5)	126 (40.0)		
Antepartum hypertension, n(%)	248 (73.8)	253 (76.0)	261 (78.6)	243 (77.1)	2.280	0.516
Antihypertensive medication						
Antepartum, n(%)						
None	113 (33.6)	120 (36.0)	124 (37.3)	84 (26.7)	15.668	0.016
Monotherapy	138 (41.1)	130 (39.0)	126 (38.0)	121 (38.4)		
Polytherapy	85 (25.3)	83 (24.9)	82 (24.7)	110 (34.9)^a^		
Postpartum two days, n(%)						
None	209 (62.2)	205 (61.6)	216 (65.1)	202 (64.1)	8.563	0.200
Monotherapy	97 (28.9)	93 (27.9)	95 (28.6)	97 (30.8)		
Polytherapy	30 (8.9)	35 (10.5)	21 (6.3)	16 (5.1)		
Postpartum hospitalization, n(%)						
None	186 (55.4)	193 (58.0)	197 (59.3)	179 (56.8)	6.743	0.345
Monotherapy	106 (31.5)	94 (28.2)	105 (31.6)	105 (33.3)		
Polytherapy	44 (13.1)	46 (13.8)	30 (9.0)	31 (9.8)		
Oxytocin						
Intrapartum, u	0.0 (0.0,2.5)	2.5 (0.0,2.5)	2.5 (0.0,2.5)^*^	2.5 (2.5,5.0)^*^	80.950	0.000
Postpartum, u	40.0 (20.0,40.0)	40.0 (20.0,40.0)	40.0 (20.0,40.0)^*^	40.0 (30.0,40.0)^*^	43.025	0.000
Fluid administration						
Postpartum two days, ml/kg/d	5.3 (4.1,5.8)	5.2 (4.0,5.8)	5.3 (4.5,5.9)	5.3 (3.7,6.1)	3.021	0.388
Postpartum hospitalization, ml/kg/d	4.7 (3.8,5.1)	4.7 (3.6,5.1)	4.8 (4.2,5.3)	4.8 (3.6,5.3)	4.439	0.218
VAS score						
30 minutes post-LEA	–	2 (1,3)	2 (1,2)	2 (1,3)	1.931	0.381
epidural catheter removal	–	3 (2,4)	3 (2,3)	3 (2,4)	1.022	0.600
Analgesia rescue, n(%)	–	31 (9.3)	49 (14.8)	43 (13.7)^b^	5.010	0.082
Lidocaine consumption, mg	–	0 (0,0)	0 (0,0)	0 (0,0)	4.469	0.107

* Compared with control group (refer to “None” for exposure of LEA), *P*<0.05.

No significant differences among the four groups in terms of age, BMI, prevalence of diabetes, type of HDP, or use of antihypertensive medication (Table 1). Compared with those who did not receive LEA, more of the participants who received medium- or long-duration LEA were primiparous (73.2% vs 51.8%, *χ*^2^ = 32.626, *P* < 0.001; 88.3% vs 51.8%, *χ*^2^ = 101.875, *P* < 0.001). Additionally, parturients who received long-duration LEA more frequently used a combination of antihypertensive medications to control antepartum hypertension (34.9% vs 25.3%, *χ*^2^ = 7.921, *P* = 0.019), yet the occurrence of antepartum hypertension in this group was comparable to that in the other three groups. In addition, women who received medium- and long-duration LEA had higher doses of oxytocin both in the intrapartum and postpartum periods than did those who did not receive LEA (Table 1). During labor, parturients who received short-duration LEA had a greater volume of fluid intake than did those in the control group did, but fluid intake during the postpartum period was comparable among the four groups (Table 1).

### LEA duration and early-onset PPHTN

Regardless of the duration of LEA exposure, both these parturients and those who did not receive LEA exhibited an increasing trend in the cumulative incidence of PPHTN within the first 5 days postpartum (S1Table). Compared with those who did not receive LEA, parturients who received long-duration LEA had significantly lower cumulative incidences of PPHTN within the initial two days postpartum (29.5% vs 41.7%, *χ*^2^ = 10.431, *P* = 0.001), whereas those who received short- or medium-duration LEA did not (S1Table). Univariate survival analysis demonstrated that long-duration LEA was associated with a lower risk of PPHTN during hospitalization (HR = 0.75, 95% CI: 0.59–0.95, *P* = 0.019), with the highest probability of remaining PPHTN-free occurring within the first 48 hours postpartum (S2Table). Compared with those who did not receive LEA, parturients who received long-duration LEA had significantly lower incidences of early-onset PPHTN (29.5% vs 41.7%, *χ*^2^ = 10.431, *P* = 0.001), whereas those who received short- or medium-duration LEA did not. After adjusting for potential confounding factors, all three models showed consistent results: long-duration LEA exposure was associated with a reduced incidence of early-onset PPHTN among parturients with HDP (Table 2).

**Table 2:** Multivariates logistic analysis of the association between LEA duration and early-onset PPHTN.

Duration of LEA	PPHTN			Model 1		Model 2		Model 3	
	n (%)	*χ* ^2^	*P*	ORs (95% CI)	*P*	ORs (95% CI)	*P*	ORs (95% CI)	*P*
None (n = 336)	140 (41.7)	11.829	0.008	Ref.	–	Ref.	–	Ref.	–
Short (n = 333)	128 (38.4)			0.85 (0.62-1.16)	0.306	0.84 (0.60-1.17)	0.295	0.79 (0.56-1.13)	0.192
Medium (n = 332)	113 (34.0)			0.75 (0.54-1.03)	0.075	0.72 (0.51-1.01)	0.057	0.73 (0.51-1.04)	0.078
Long (n = 315)	93 (29.5)^*^			0.63 (0.45-0.89)	0.008	0.53 (0.37-0.76)	< 0.001	0.55 (0.38-0.81)	0.002

*Compared with control group (refer to “None”), *P* < 0.05.

Model 1: ORs were adjusted for maternal age, BMI, preterm birth, primipara.

Model 2: ORs were adjusted for Model 1 plus type of HDP, presence of antepartum hypertension, and antepartum antihypertensive therapy.

Model 3: ORs were adjusted for Model 2 plus postpartum fluid administration and antihypertensive therapy.

In the sensitivity analysis limited to participants with documented antepartum hypertension who received multiple antihypertensive medications, LEA exposure across all three duration categories was significantly associated with a reduced incidence of early-onset PPHTN when compared to women who did not receive LEA (Table 3). We then reanalyzed our data after excluding participants who had antihypertensive therapy during postpartum period. The risk of early-PPHTN was slightly attenuated in the multivariable model among parturients who received medium- and long-duration LEA (Table 3). However, when the follow-up period was extended to five days postpartum, the association between long-duration LEA and reduced incidence of PPHTN observed in the main analysis was attenuated and did not reach statistical significance (Table 3). Stratified analyses revealed no significant difference in the risk of early-onset PPHTN between parturients with advanced maternal age, multiparity, obesity, or PE who received long-duration LEA and those who received shorter durations of LEA (Table 4).

**Table 3:** Sensitivity analyses examining the association between LEA duration and early-onset PPHTN.

	Participants had antepartum hypertension and received multiple antepartum antihypertensive medications (n = 320)		Participants without postpartum antihypertensive medications (n = 755)		PPHTN incidence within 5 days postpartum (n = 1316)	
	n (%)	ORs (95% CI)^a^	n (%)	ORs (95% CI)^b^	n (%)	ORs (95% CI)^c^
None	61 (82.4)	Ref.	55 (29.6)	Ref.	150 (44.6)	Ref.
Short	49 (62.8)	0.28 (0.12-0.64)	44 (22.8)	0.75 (0.46-1.21)	145 (43.5)	0.94 (0.66-1.34)
Medium	44 (59.5)	0.32 (0.14-0.72)	37 (18.8)	0.51 (0.31-0.85)	132 (39.8)	0.86 (0.60-1.24)
Long	45 (47.9)	0.21 (0.09-0.46)	31 (17.3)	0.44 (0.26-0.75)	121 (38.4)	0.72 (0.50-1.06)
*χ* ^2^	21.337	17.756	9.714	11.262	3.577	3.044
*P*	< 0.001	< 0.001	0.021	0.010	0.311	0.385

^a^ORs were adjusted for maternal age, BMI, preterm birth, primipara, type of HDP, postpartum fluid administration and postpartum antihypertensive therapy.

^b^ORs were adjusted for maternal age, BMI, preterm birth, primipara, type of HDP, presence of antepartum hypertension, antepartum antihypertensive therapy, and postpartum fluid administration.

^c^ORs were adjusted for maternal age, BMI, preterm birth, primipara, type of HDP, presence of antepartum hypertension, antepartum antihypertensive therapy, postpartum fluid administration and postpartum antihypertensive therapy.

**Table 4:** Stratified Analyses of the Association Between LEA and early-onset PPHTN.

	Maternal age		Parity		Obesity		HDP	
	<35 years, (n = 1088)	≥35 years, (n = 228)	Primipara, (n = 869)	Multipara, (n = 447)	< 28 kg/m^2^, (n = 958)	≥ 28 kg/m^2^, (n = 358)	GH, (n = 790)	PE, (n = 526)
None (n = 336)	Ref.	Ref.	Ref.	Ref.	Ref.	Ref.	Ref.	Ref.
Short (n = 333)	0.70 (0.47-1.04)	1.72 (0.74-3.97)	0.84 (0.51-1.39)	0.89 (0.53-1.49)	0.77 (0.51-1.17)	0.90 (0.46-1.78)	0.90 (0.57-1.41)	0.75 (0.42-1.34)
Medium (n = 332)	0.73 (0.49-1.08)	0.81 (0.32-2.03)	0.80 (0.51-1.26)	0.67 (0.36-1.24)	0.75 (0.49-1.14)	0.75 (0.37-1.52)	0.71 (0.45-1.14)	0.85 (0.48-1.51)
Long (n = 315)	0.54 (0.36-0.81)^*^	0.47 (0.16-1.33)	0.56 (0.35-0.88)^*^	0.55 (0.24-1.26)	0.48 (0.31-0.75)^*^	0.71 (0.32-1.56)	0.41 (0.24-0.69)	0.81 (0.45-1.47)
*χ* ^2^	8.950	6.158	7.142	3.083	10.954	0.96	13.274	1.048
*P*	0.030	0.104	0.068	0.379	0.012	0.811	0.004	0.790

ORs were adjusted for the same covariates as Model 3, excluding the corresponding stratified variable.

* Compared with control group (refer to “None”), *P* < 0.05.

### Postpartum requirement for antihypertensive medication

No significant differences among the four groups in terms of the use of antihypertensive medication (Table 1), while the frequency of hypertension during the postpartum stay was significantly lower in parturients who received medium-duration and long-duration LEA than in those who did not receive LEA (0.00 [0.00,0.17] vs 0.00 [0.00,0.33], H = 71.412, P = 0.041, and 0.00 [0.00,0.17] vs 0.00 [0.00,0.33], H = 74.364, P = 0.032, respectively).

In parturients with GH, the duration of LEA exposure did not significantly affect the use of antihypertensive medications or the requirement for polytherapy during the postpartum hospital stay (S3Table). Compared with parturients with GH, parturients with PE had increased postpartum use of antihypertensive medications (52.3% vs 36.2%, *χ*^2^ = 33.379, *P* < 0.001), and a greater proportion required polytherapy for blood pressure control (18.8% vs 6.6%, *χ*^2^ = 46.565, *P* < 0.001). Notably, although the duration of LEA exposure did not reduce the postpartum use of antihypertensive medications in parturients with PE, the requirement for polytherapy was found to be lower in parturients exposed to medium- and long-duration LEA than in those exposed to short-duration LEA and those who did not receive LEA (S3 Table).

## Discussion

Currently, a significant number of parturients are anticipated to receive LEA, and the effects of LEA during delivery for parturients with HDP are well studied [18]. However, few investigations have assessed the potential impact of LEA on PPHTN in parturients diagnosed with HDP. The survival analysis in our study demonstrated that time-dependent exposure to LEA was associated with a reduced risk of developing PPHTN during hospitalization, with the highest probability of remaining PPHTN-free occurring within the first 48 hours postpartum. Given that this period also represents a time of heightened risk for severe hypertensive complications, such as eclampsia and stroke, we selected this critical window to evaluate the potential association between LEA and the incidence of PPHTN [19].

In line with previous findings, this retrospective cohort study revealed a progressive rise in the cumulative incidence of PPHTN during the early postpartum period, with over one-third of women diagnosed with HDP developing hypertension within the first 48 hours after delivery [20]. This heightened risk during the early postpartum phase is likely attributable to a combination of physiological changes, including the administration of large volumes of intravenous fluids during labor, the abrupt cessation of pregnancy-induced vasodilation, and the rapid mobilization of extravascular fluid following delivery [21,22]. Notably, our analysis revealed that parturients with HDP who received long-duration LEA had a significantly lower risk of developing early-onset PPHTN, independent of other confounding factors, as demonstrated by multivariable logistic regression analysis.

To account for potential confounding by disease severity, we conducted a sensitivity analysis restricted to parturients with documented antepartum hypertension and severe hypertensive phenotypes, defined as those requiring antihypertensive polytherapy during pregnancy. The results showed that the ORs were significantly reduced across all LEA duration groups, suggesting that the main study findings may have been underestimated. This could be because some women with milder antepartum hypertension had a lower likelihood of developing PPHTN within 48 hours postpartum, potentially masking the protective effect of LEA in this subgroup. Furthermore, among women who did not receive antihypertensive medications postpartum, the ORs across all groups were largely consistent with the main effect, supporting an independent protective role of long-duration LEA in reducing the risk of early-onset PPHTN. However, when the follow-up period was extended to five days postpartum, the ORs increased significantly across all groups, suggesting that the protective effect of LEA was no longer evident beyond this timeframe. This finding is consistent with our hypothesis that the beneficial impact of prolonged LEA is primarily attributable to its continued effects during the early postpartum period, which may not persist beyond 48 hours after delivery.

In addition, our study showed that the use of LEA was not associated with a reduced need for postpartum antihypertensive medications among women with HDP. However, women who received long-duration LEA were less likely to develop hypertension during the postpartum hospitalization period. Furthermore, the need for combined antihypertensive therapy postpartum appeared to be lower among parturients with PE who received LEA. These findings suggests that long-duration LEA, when combined with antihypertensive therapy, may offer improved blood pressure control in women with HDP, indicating a potential beneficial role of LEA use in the postpartum management of these patients [23].

According to previous studies, the perceived benefits of LEA in women with HDP have primarily been attributed to its analgesic effects during labor, which may have led to an underestimation of its hemodynamic impact [24]. Despite this underappreciated role, emerging perspectives suggest that epidural anesthesia may offer more than pain relief, with some authors proposing it as a potential therapeutic strategy for preeclampsia patients with severe features [25,26]. LEA exerts its primary hemodynamic effect through blockade of sympathetic nerve activity, resulting in peripheral vasodilation and a subsequent reduction in total vascular resistance [27]. This mechanism may be particularly effective in managing vasoconstrictive hypertension, which represents one of the two principal hemodynamic pathways contributing to maternal hypertension [28]. It is important to note, however, that the blood pressure-lowering effect of LEA may be limited in certain subgroup. Our study showed that even long-duration LEA did not significantly reduce the risk of early-onset PPHTN among advanced maternal age, obese, or PE parturients. These findings suggest that alternative or additional strategies may be needed to manage postpartum blood pressure in these high-risk groups, and highlight the importance of individualized postpartum monitoring and antihypertensive management [29].

To the best of our knowledge, our study is one of the largest clinical investigations specifically designed to evaluate the potential impact of LEA on PPHTN in parturients diagnosed with HDP. Several limitations of our study should be acknowledged. First, parturients in our study underwent routine blood pressure monitoring every 4–6 hours. Those with elevated readings were placed under more frequent monitoring. This clinical practice resulted in a higher number of blood pressure measurements among women who developed PPHTN. Conversely, it may have led to an under-ascertainment of PPHTN in women without the condition, as fewer measurements were obtained in this group. In addition, as there were no established clinical studies to guide the categorization of LEA exposure duration, this study stratified participants based on tertiles of LEA duration observed in the cohort. Therefore, our findings primarily reflect an association between exposure duration and the outcome. Our sensitivity analyses also suggested that optimal cutoff values for LEA duration may vary across different risk groups and warrant further investigation. Moreover, our centre used a standardized concentration of ropivacaine for LEA, future studies should consider the potential impact of varying local anesthetic concentrations on postpartum hypertensive outcomes.

## Conclusion

A longer duration of LEA was associated with a reduced risk of early-onset PPHTN among women with HDP. However, this association did not reach statistical significance in subgroups of parturients with advanced maternal age, obesity, or PE.

## Supporting information

S1 Fig Flow Diagram. BP indicates blood pressure; BMI, body mass index; HDP, hypertensive disorders of pregnancy; PPHTN, postpartum hypertension.
(TIF)

S1 Table The cumulative incidence of PPHTN within the postpartum hospitalization among different durations of LEA exposure. (DOCX)

S2 Table Survival table and univariate survival analysis between LEA duration and PPHTN during hospitalization. (DOCX)

S3 Table Comparison of the necessity for antihypertensive medication among the different durations of LEA exposure. (DOCX)

S1 File STROBE Checklist. (DOCX)

S2 File Study data. (XLSX)
