# Supplementary figures and images for: The association between labor epidural analgesia and early-onset postpartum hypertension among parturients with hypertensive disorders of pregnancy: A retrospective cohort study
Source: PLoS One. 2025 Aug 18;20(8):e0325476. doi: 10.1371/journal.pone.0325476 (PMC12360508; doi:10.1371/journal.pone.0325476)

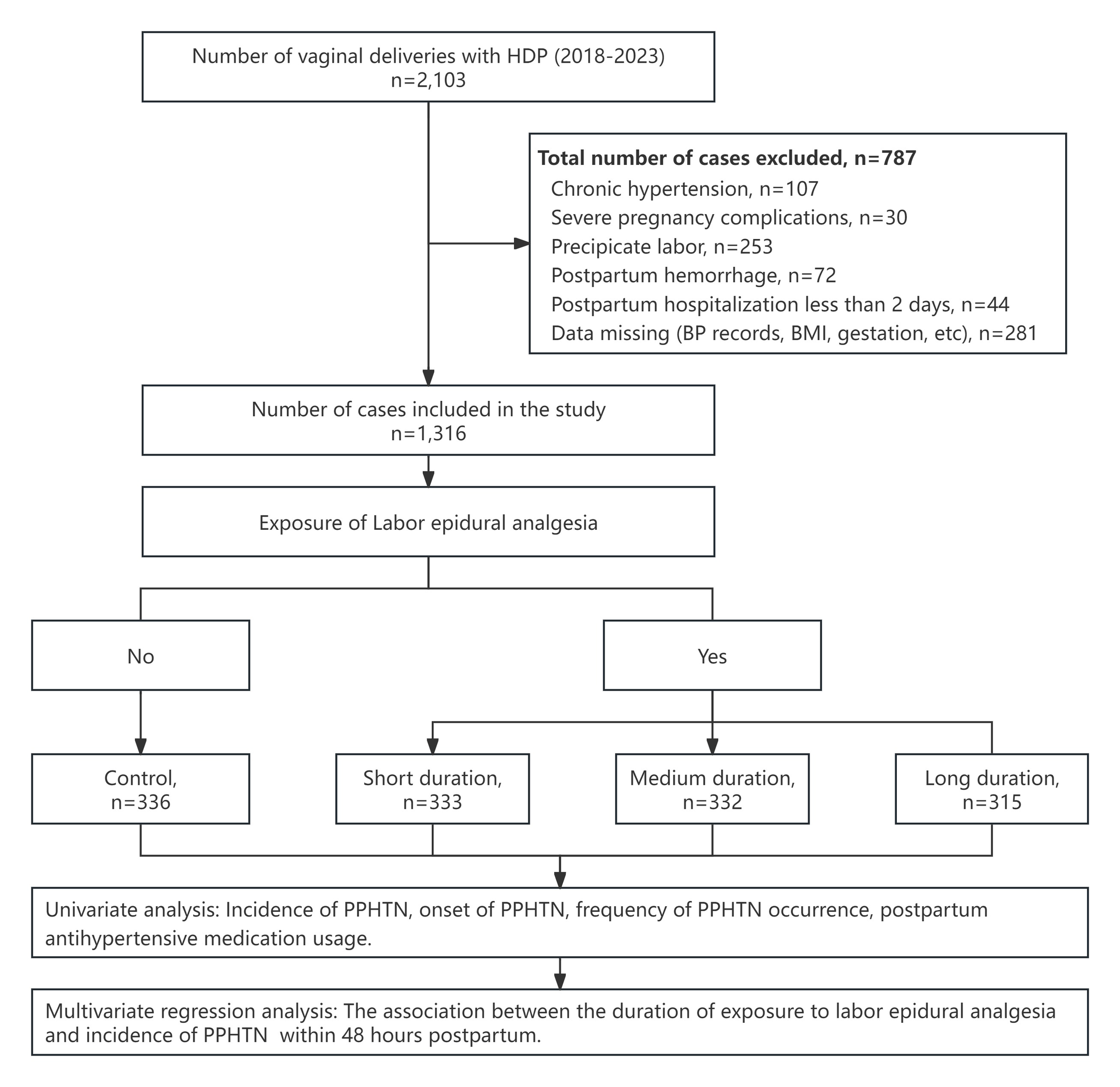

Supplement: S1 Fig — BP indicates blood pressure; BMI, body mass index; HDP, hypertensive disorders of pregnancy; PPHTN, postpartum hypertension. (TIF) [file pone.0325476.s001.tif]
