# Supplementary material for: The association between labor epidural analgesia and early-onset postpartum hypertension among parturients with hypertensive disorders of pregnancy: A retrospective cohort study
Source: PLoS One. 2025 Aug 18;20(8):e0325476. doi: 10.1371/journal.pone.0325476 (PMC12360508; doi:10.1371/journal.pone.0325476)
Supplement: S1 Table — (DOCX) [file pone.0325476.s002.docx]

| Table S1. The cumulative incidence of PPHTN within the postpartum hospitalization among different durations of LEA exposure | | | | | | |
| --- | --- | --- | --- | --- | --- | --- |
| Exposure of LEA | Cumulative incidence of PPHTN, n(%) | | | | | |
|  | Postpartum day 1 | Postpartum day 2 | Postpartum day 3 | Postpartum day 4 | Postpartum day 5 | Overall |
| None (n=336) | 112 (33.3) | 140 (41.7) | 146 (43.5) | 149 (44.3) | 150 (44.6) | 150 (44.6) |
| Short (n=333) | 101 (30.3) | 128 (38.4) | 143 (42.9) | 144 (43.2) | 145 (43.5) | 146 (43.8) |
| Medium (n=332) | 82 (24.7) | 113 (34.0) | 127 (38.3) | 132 (39.8) | 132 (39.8) | 132 (39.8) |
| Long (n=315) | 74 (23.5)^*^ | 93 (29.5)^*^ | 111 (35.2) | 118 (37.5) | 121 (38.4) | 124 (39.4) |
| χ^2^ | 10.598 | 11.829 | 8.265 | 4.045 | 3.577 | 3.010 |
| *P* | 0.014 | 0.008 | 0.099 | 0.257 | 0.311 | 0.390 |
| ^*^ Compared with control group (refer to “None”), *P*<0.05. | | | | | | |
