# Supplementary material for: The association between labor epidural analgesia and early-onset postpartum hypertension among parturients with hypertensive disorders of pregnancy: A retrospective cohort study
Source: PLoS One. 2025 Aug 18;20(8):e0325476. doi: 10.1371/journal.pone.0325476 (PMC12360508; doi:10.1371/journal.pone.0325476)
Supplement: S2 Table — (DOCX) [file pone.0325476.s003.docx]

| Table S2. Survival table and univariate survival analysis between LEA duration and PPHTN during hospitalization | | | | | | | |
| --- | --- | --- | --- | --- | --- | --- | --- |
| Exposure of LEA | Probability of survival (%) | | | | | COX (Unvariate) | |
|  | Postpartum day 1 | Postpartum day 2 | Postpartum day 3 | Postpartum day 4 | Postpartum day 5 | HR (95% CI) | *P* |
| None (n=336) | 65.2 | 55.4 | 51.8 | 44.4 | 44.4 | Ref. | - |
| Short (n=333) | 69.7 | 59.9 | 44.3 | 41.1 | 30.8 | 1.00 (0.80-1.26) | 0.998 |
| Medium (n=332) | 75.3 | 64.2 | 52.2 | 37.4 | 37.4 | 0.85 (0.68-1.08) | 0.186 |
| Long (n=315) | 76.5 | 69.5 | 56.5 | 41.4 | 27.6 | 0.75 (0.59-0.95) | 0.019 |
