# Supplementary material for: The association between labor epidural analgesia and early-onset postpartum hypertension among parturients with hypertensive disorders of pregnancy: A retrospective cohort study
Source: PLoS One. 2025 Aug 18;20(8):e0325476. doi: 10.1371/journal.pone.0325476 (PMC12360508; doi:10.1371/journal.pone.0325476)
Supplement: S3 Table — (DOCX) [file pone.0325476.s004.docx]

| Table S3. Comparison of the necessity for antihypertensive medication among the different durations of LEA exposure | | | | | | | | | | | | |
| --- | --- | --- | --- | --- | --- | --- | --- | --- | --- | --- | --- | --- |
|  | Gestation hypertension | | | | | | Preeclampsia | | | | | |
|  | None  (n=197) | Short  (n=203) | Medium  (n=201) | Long  (n=189) | *χ*^2^ | *P* | None  (n=139) | Short  (n=130) | Medium  (n=131) | Long  (n=126) | *χ*^2^ | *P* |
| Antepartum, n(%) | | | | | | | | | | | | |
| None | 76 (38.6) | 90 (44.3) | 84 (41.8) | 57 (30.2) | 16.660 | 0.011 | 37 (26.6) | 30 (23.1) | 40 (30.5) | 27 (21.4) | 7.759 | 0.256 |
| Monotherapy | 87 (44.2) | 77 (37.9) | 71 (35.3) | 76 (40.2) |  |  | 51 (36.7) | 53 (40.8) | 55 (42.0) | 45 (35.7) |  |  |
| Polytherapy | 34 (17.3) | 36 (17.7) | 46 (22.9) | 56 (29.6)a |  |  | 51 (36.7) | 47 (36.2) | 36 (27.5) | 54 (42.9) |  |  |
| Postpartum two days, n(%) | | | | | | | | | | | | |
| None | 136 (69.0) | 144 (70.9) | 136 (67.7) | 128 (67.7) | 4.894 | 0.557 | 73 (52.5) | 61 (46.9) | 80 (61.1) | 74 (58.7) | 19.446 | 0.003 |
| Monotherapy | 57 (28.9) | 51 (25.1) | 53 (26.4) | 54 (28.6) |  |  | 40 (28.8) | 42 (32.3) | 42 (32.1) | 43 (34.1) |  |  |
| Polytherapy | 4 (2.0) | 8 (3.9) | 12 (6.0) | 7 (3.7) |  |  | 26 (18.7) | 27 (20.8) | 9 (6.9)^*^ | 9 (7.1)^*^ |  |  |
| Postpartum hospitalization, n(%) | | | | | | | | | | | | |
| None | 122 (61.9) | 140 (69.0) | 126 (62.7) | 116 (61.4) | 8.298 | 0.217 | 64 (46.0) | 53 (40.8) | 71 (54.2) | 63 (50.0) | 22.143 | 0.001 |
| Monotherapy | 67 (34.0) | 52 (25.6) | 57 (28.4) | 58 (30.7) |  |  | 39 (28.1) | 42 (32.3) | 48 (36.6) | 47 (37.3) |  |  |
| Polytherapy | 8 (4.1) | 11 (5.4) | 18 (9.0) | 15 (7.9) |  |  | 36 (25.9) | 35 (26.9) | 12 (9.2)^*^ | 16 (12.7)^*^ |  |  |
| ^*^ Compared with control group (refer to “None” for exposure of LEA), *P*<0.05. | | | | | | | | | | | | |
